# Features of branch occlusive disease-type intracranial atherosclerotic stroke in young patients

**DOI:** 10.1186/s12883-018-1089-1

**Published:** 2018-06-20

**Authors:** Zhang-Ning Zhao, Xiao-Lin Li, Jin-Zhi Liu, Zhi-Ming Jiang, Ai-Hua Wang

**Affiliations:** 1 Department of Neurology, Affiliated Qianfoshan Hospital of Shandong University, 66 Jingshi Road, Jinan, 250014 Shandong province People’s Republic of China; 2 Department of Critical Care Medicine, Affiliated Qianfoshan Hospital of Shandong University, 66 Jingshi Road, Jinan, 250014 Shandong province People’s Republic of China

**Keywords:** Stroke, Young adult, Branch occlusive disease, MRI

## Abstract

**Background:**

Young ischemic stroke patients are common while classification and analysis based upon imaging characteristics are rarely reported. We intend to compare the clinical and MRI characteristics of cerebral stroke induced by intracranial atherosclerosis between young patients with branch occlusive disease (BOD) and those with non-branch occlusive disease (non-BOD) or small artery disease (SAD).

**Methods:**

A total of 151 subjects with acute infarction within the middle cerebral artery (MCA) territory were included and patients with ipsilateral internal carotid artery stenosis or cardioembolism were excluded. Based on the distribution characteristics of infarction and the presence of ipsilateral MCA stenosis, the patients were divided into three groups: BOD-striatocapsular area infarction with ipsilateral MCA stenosis; non-BOD -infarction size exceeds the striatocapsular area and accompanied by ipsilateral MCA stenosis; SAD. The clinical and MCA stenosis characteristics of the three groups were compared.

**Results:**

The number of BOD patients with hypertension was significantly higher than that of SAD (92.9% vs 53.7%, *p* = 0.000) and non-BOD (92.9% vs 57.1%, *p* = 0.001); subjects with smoking history significantly exceeded that of SAD (50% vs 26.9%, *p* = 0.03) and subjects with family history of cardiovascular disease was significantly less than that of non-BOD (14.3% vs 41.1%). Baseline NIHSS scores and mRS scores at discharge in patients with BOD were significantly lower than those with non-BOD (*p* = 0.000, *p* = 0.001). Majority of patients in non-BOD group displayed severe MCA stenosis (39 cases, 69.6%) while that in BOD group displayed mild stenosis (26 cases, 92.9%), and the difference was statistically significant (*p* = 0.000). Compared with non-BOD group, the stenosis in BOD group located at a relatively distal end in the M1 segment of MCA (S/M1, 58% vs 40%, *p* = 0.000) and was more localized (stenosis level/ (SL/M1), 1.86 (1.35–2.6) vs 2.9 (2.0–5.0), *p* = 0.002).

**Conclusion:**

BOD in young patients with ischemic stroke induced by intracranial atherosclerosis is not rare (33.3%) and its clinical manifestations and prognosis are similar to those of SAD. This may be related to the mild localized stenosis at the distal end in the M1 segment of MCA. Control of hypertension might play a positive role in secondary prevention.

## Background

Intracranial atherosclerotic stroke (ICAS) is quite common in Asian populations, and can be found in up to 56% of Chinese patients with ischemic stroke [1]. It is currently believed that the pathogenesis includes in situ thrombotic occlusion, artery-to-artery embolism, hypoperfusion and branch artery occlusion [2]. BOD refers to the subcortical infarction resulting from the blockage of artery orifice due to the atherosclerotic plaques of carrier artery. And a study using high-resolution magnetic resonance imaging (MRI) reveals the unique vascular remodeling and plaque characteristics of BOD [3].

We found in clinical practice that young stroke patients are common and its pathogenesis is diverse, but atherosclerosis remains one of the major causes. Imaging results tend to detect intracranial arterial lesions, particularly stenosis or occlusion of the MCA, and BOD may be one of the major types of stroke. To the best of our knowledge, there is currently limited comparative study of young patients with BOD and non-BOD stroke. In this study, we will preliminarily assess the incidence of BOD in young stroke patients and compare the clinical features (especially risk factors) among BOD, non-BOD and SAD patients, and the characteristics of MCA stenosis between BOD and non-BOD patients.

## Methods

### Objects of study

Acute cerebral infarction patients diagnosed by the MRI Unit of Affiliated Qianfoshan Hospital of Shandong University between July 2014 and July 2016 were selected as objects of study.

Inclusion criteria: (1) acute or subacute onset, age <55 years; (2) cerebral MRI scans included diffusion weighted image (DWI), and DWI indicated high signal intensity within the MCA territory; (3) with or without symptoms and signs of neurological deficits, such as aphasia, paralysis, paresthesia, dysarthria, ataxia, etc.; (4) informed consent of patients and their families.

Exclusion criteria: (1) infarction with relevant ipsilateral internal carotid artery stenosis; (2) stroke due to non-atherosclerosis cause, such as dissection or moyamoya disease; (3) incomplete patient data, such as the lack of NIHSS score, mRS score or MRA examination.

Based on the cerebral DWI distribution and the presence of ipsilateral MCA stenosis, the patients were divided into three groups: (1) branch occlusive disease (BOD) group: patients with striatocapsular area infarction and relevant MCA stenosis; (2) non-BOD group: infarction size exceeds the striatocapsular area (such as the cortical area) and accompanied by stenosis at ipsilateral MCA; (3) small artery disease (SAD) group: deep infarction without stenosis at ipsilateral MCA.

### Data collection

The patients’ essential clinical data (including name, gender, age, previous stroke or transient ischemic attack (TIA) history, history of diabetes, heart disease with coronary atherosclerosis, hyperlipidemia, hypertension, smoking, and family history of cardiovascular and cerebrovascular disease), NIHSS scores and mRS scores were collected.

Definitions of vascular risk factors were in accordance with the following criteria:

1. Stroke or transient ischemic attack (TIA) was deemed present when the patient was previously diagnosed as stroke or TIA by a neurologist according to the 2014 Chinese guidelines for diagnosis and treatment of acute ischemic stroke [4].
2. Diabetes was defined as fasting plasma glucose ≥ 7.8 mmol/L or random plasma glucose ≥ 11.1 mmol/L or glucose tolerance test 2 h ≥ 11.1 mmol/L, or the patient was previously diagnosed as diabetes by endocrinologists and/or receiving hypoglycemic drug treatment.
3. Heart disease with coronary atherosclerosis was considered present if the patient was previously diagnosed as coronary atherosclerotic heart disease by cardiologists, with history of angina or myocardial infarction [5, 6].
4. Hyperlipidemia was defined as being consistent with either of the following:total cholesterol (TC) ≥ 6.22 mmol/L; or low density lipoprotein (LDL-C) ≥ 4.14 mmol/L; or triglyceide (TG) ≥ 2.26 mmol/L; or previously diagnosed as hyperlipidemia by clinicians according to the Chinese guidelines for prevention and treatment of dyslipidemia [7].
5. Hypertension was regarded as being present if all three times of blood pressure measurements of the patient not on a single day, without taking antihypertensive drugs, indicate systolic blood pressure ≥ 140 mmHg and (or) diastolic blood pressure ≥ 90 mmHg, or the patient was previously diagnosed as hypertension by clinicians and antihypertensive drugs are being administered regardless of the current blood pressure level.
6. Smoking history was defined as a duration of cigarette smoking exceeding 6 months and a daily amount being greater than one.

### Imaging analysis

All participants underwent MRI on a 3-T system using a 8-channel phased-array head coil (Siemens AG Healthcare, Erlanger, Germany). Scanning parameters: axial T2 (repetition time 4500 ms, echo time 90 ms); axial T1 (repetition time 2000 ms, echo time 9.0 ms); DWI (repetition time 3300 ms, echo time 90 ms); slice thickness 5 mm, interslice gap 1.5 mm; 3-dimensional, time-of-flight MRA (repetition time 25 ms, echo time 3.5 ms, slice thickness 0.45 mm, 80 slices, 20° flip angle, a 880 × 450 matrix, and field of view 170 mm).

**Fig. 1:**
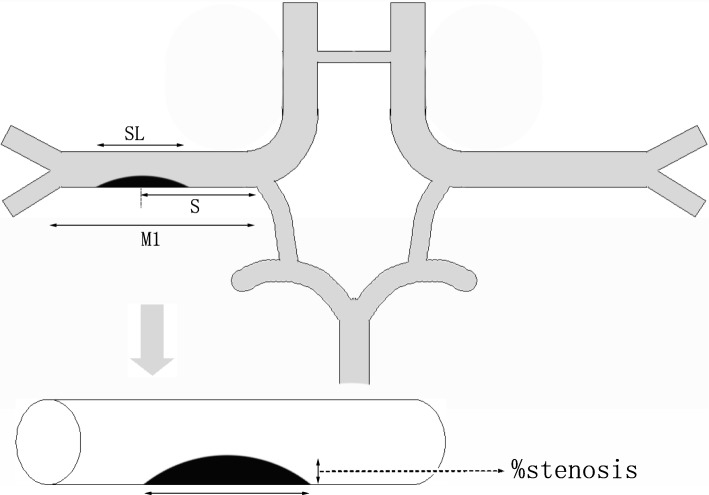
Profile of MCA stenosis. As shown in the figure, S represents the distance from the beginning of MCA to the center of stenosis, M1 represents the distance from the beginning of MCA to the point of bifurcation, and SL represents the stenosis length. Percent diameter stenosis (%stenosis) was calculated using the WASID method [8]

The stenosis of MCA was measured separately by two experienced neurologists according to the methods applied in previous studies [8] and recorded when reaching a consensus. If the two neurologists’ results did not agree, a final decision was made by a third neurologist. The stenosis of MCA is divided into five levels: no stenosis, >30, >50, 70–99%, and occlusion. Two experienced neurologists separately measured the distance from the beginning of MCA to the center of stenosis (S), from the beginning of MCA to the point of bifurcation (M1) and the stenosis length (SL) (as shown in Fig. 1), agreed and recorded. When the offending MCA was occluded, the length of the contralateral M1 of MCA was measured instead and SL was recorded as missing. S/M1 was adopted to evaluate stenosis location, SL/M1 ratio for evaluating the scope of stenosis and stenosis level/ (SL/M1) for the morphology of stenosis [9]. Further comparative analysis was conducted on the risk factors, imaging features, short-term prognosis, etc. of three groups of patients.

### Statistical analysis

Normally distributed data of continuous variables was expressed as mean ± standard deviation and non-normally distributed data of continuous variables was expressed as median (interquartile range); categorical variables are expressed as frequency or percentage. Comparison between groups with continuous variables of normal distribution were analyzed by t-test and those of non-normal distribution by Wilcoxon rank-sum test; X^2^ test or Fisher’s exact test was used to compare the categorical variables between groups. The difference was statistically significant at *P* < 0.05. Univariate and multivariate Logistic regression analysis was used for the imaging prognostic factors of prognosis. All statistical analyses were conducted using software SPSS 23.0.

## Results

**Fig. 2:**
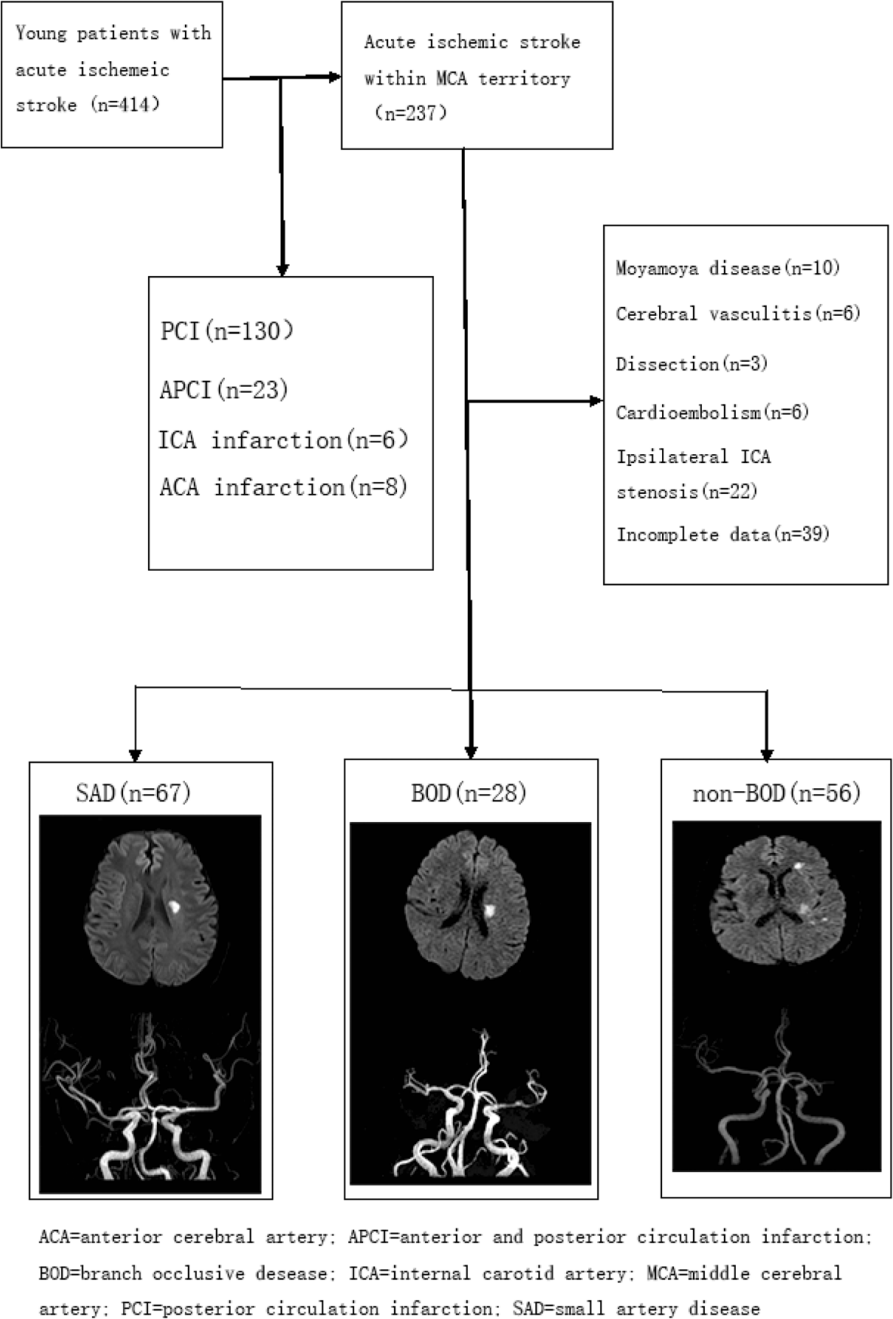
Patients selection. Of 237 patients with acute MCA infarctions, 151 patients were included and divided into three groups: SAD, BOD, and non-BOD. ACA = anterior cerebral artery; APCI = anterior and posterior circulation infarction; BOD = branch occlusive disease; ICA = internal carotid artery; MCA = middle cerebral artery; PCI = posterior circulation infarction; SAD = small artery disease

Of the 414 young ischemic stroke patients included in this study, 237 were eligible for MCA infarction (Fig. 2), excluding 25 stroke cases of other causes (including 10 cases of moyamoya disease, six cases of cerebral arteritis, three cases of cerebral artery dissection, six cases of cardiac infarction), 39 cases with incomplete data and 22 cases with ipsilateral internal carotid artery stenosis. The remaining 151 patients were divided into SAD group (67 cases), BOD group (28 cases) and non-BOD group (56 cases), according to the characteristics of infarction site and MCA stenosis. Table 1 shows the general analysis.

**Table 1:** Comparison of general charactors among the SAD, BOD and non-BOD groups

	SAD	BOD	non-BOD	*P* value		
	*n* = 67	*n* = 28	*n* = 56	Among groups	BOD vs SAD	BOD vs non-BOD
Age, x ± s	49.5 ± 4.0	48.5 ± 6.5	47.9 ± 6.3	0.271	0.434	0.621
Male, *n* (%)	38 (56.7)	24 (85.7)	48 (85.7)	0.000	0.007	1.000
Risk factors, *n* (%)						
Stroke	5 (7.5)	2 (7.1)	12 (21.4)	0.042	0.957	0.098
Hypertension	36 (53.7)	26 (92.9)	32 (57.1)	0.001	0.000	0.001
Diabetes	17 (25.4)	10 (35.7)	16 (28.6)	0.595	0.308	0.504
Current smoking	18 (26.9)	14 (50)	25 (44.6)	0.043	0.03	0.643
Family CAD history	26 (38.8)	4 (14.3)	23 (41.1)	0.013	0.056	0.005
Baseline NIHSS score median (IQR)	3 (1–5)	2 (1–4)	6 (3–11)	0.000	0.603	0.000
TOAST classication, *n* (%)				0.000	0.302	0.021
LAD	0 (0)	2 (7.14)	49 (87.5)			
SAD	24 (35.8)	11 (39.3)	0 (0)			
UE	43 (64.2)	15 (53.6)	7 (12.5)			
mRS core on discharge				0.000	0.454	0.001
0–2分, *n* (%)	53 (79.1)	24 (85.7)	27 (48.2)			

*Abbreviations*: *BOD* branch occlusive disease, *SAD* small artery disease, *LAD* large artery disease, *CAD* coronary artery disease, *IQR* interquartile range, *NIHSS* National Institutes of Health Stroke Scale, *TOAST* Trial of Org 10,172 in Acute Stroke Treatment, *UE* undetermined etiology

There was no significant difference in age among the three groups. Percentage of male in BOD and non-BOD groups was 85.7%, which was significantly higher than that of SAD group (*p* = 0.000). The prevalence of intercurrent hypertension in BOD patients was significantly higher than that in SAD patients (92.9% vs 53.7%, *p* = 0.000) and non-BOD patients (92.9% vs 57.1%, *p* = 0.001); patients with smoking history in BOD group significantly exceeded SAD group (50% vs 26.9%, *p* = 0.03) and family history of cardiovascular and cerebrovascular diseases was significantly less than that of non-BOD group (14.3% vs 41.1%). Past history of stroke and diabetes history presented no significant difference between the three groups. Baseline NIHSS scores and mRS scores at discharge in BOD group were not significantly different from those in SAD group but were significantly lower than those in non-BOD group (*p* = 0.000, *p* = 0.001, respectively). In terms of the wide difference of classifying patients to BOD group according to radiographic results or TOAST classification, only two patients (7.14%) among the 28 patients with BOD met the TOAST classification for BOD diagnosis and 11 (39.3%) patients met the TOAST classification for SAD diagnosis, and over half of patients met the TOAST classification for unexplained cause.

See features of MCA stenosis in Table 2. MCA stenosis in non-BOD group was more severe and most cases presented severe stenosis (>70%, 39 cases, 69.6%) including 28 cases of MCA occlusion. In comparison, the majority of patients in BOD group showed mild MCA stenosis (≤50%, 26 cases, 92.9%) and the difference was statistically significant (*p* = 0.000). However, the relative MCA stenosis length (SL/M1) of BOD group and non-BOD group was not significantly different (18% vs 19%, *p* = 0.529) after excluding patients with MCA occlusion. Compared with non-BOD group, the stenosis in BOD group located at a relatively distal end in the M1 segment of MCA (S/M1, 58% vs 40%, *p* = 0.000) and was more localized (stenosis level/ (SL/M1), 1.86 (1.35–2.6) vs 2.9 (2.0–5.0), *p* = 0.002).

**Table 2:** Comparison of MCA stenosis features between BOD and non-BOD groups

	BOD *n* = 28	non-BOD *n* = 56	*P* value
MCA stenosis, degree, *n* (%)			0.000
≤50%	26 (92.9)	15 (26.8)	
50–70%	2 (7.1)	2 (3.6)	
>70%	0 (0)	39 (69.6)	
S/M1, x ± s	0.58 ± 0.18	0.40 ± 0.19	0.000
SL/M1, x ± s	0.18 ± 0.07	0.19 ± 0.05	0.529
%stenosis/(SL/M1) median (IQR)	1.86 (1.35–2.6)	2.9 (2.0–5.0)	0.002

*Abbreviations*: *MCA* middle cerebral artery, *IQR* interquartile range

## Discussion

Our study showed that BOD type accounts for one-third in young patients with ICAS while previous studies showed that this proportion is nearly half in stroke patients of all age groups [10]. However, for a variety of reasons, researchers may have underestimated the incidence of BOD.

First, TOAST stroke classification did not explicitly propose BOD and the diagnosis of stroke with large-artery atherosclerosis requires stenosis ≥50% at related artery [11], while previous studies showed that nearly 60% of BOD patients had only mild cerebral artery stenosis [10] and even up to 92.9% of young BOD patients in this study had mild stenosis. This may be the reason why so many patients, especially young patients experienced missed diagnosis.

In addition, the classification criteria defined an infarct size less than 15 mm [11] or 20 mm [12] as SAD. Despite of the different pathogenesis, BOD often leads to lacunar infarction and previous studies [13, 14] and this study suggest that the clinical manifestations of BOD and SAD are very similar. As a result, BOD is easily confused with SAD in the absence of vascular imaging. This study showed that only 7.14% of young patients conform to the diagnostic criteria of BOD while up to 39.3% conform to those of SAD according to the TOAST classification.

Many perforating branches are rooted in intracranial artery and mild stenosis may occlude their orifices, leading to stroke in the absence of plaque rupture or thrombosis. But clinicians may sometimes pay insufficient attention to mild stenosis and consider it irrelevant to the incidence. A follow-up study of patients with ischemic stroke showed that, in patients diagnosed with SAD at the first time of stroke, clinicians often found intracranial stenosis in them during stroke recurrences [1]. Furthermore, studies have found that in patients with pontine infarction while vascular imaging showed no abnormalities in the basilar artery, additional high-resolution MRI confirmed that 42% of them had basilar artery plaques [15]. Since high resolution MRI is not widely used in clinical practice, we are most likely to have missed a number of patients with intracranial atherosclerotic stroke and incorrectly classified them as undetermined etiology.

Although the present study shows similar short-term prognosis and little difference in neurological impairment between BOD and SAD in the early stages of onset, it is likely that the BOD is closely related to stroke progression and recurrence [16]. Since young patients have long life expectancy and intense social function requirements, multiple recurrence will inevitably lead to the decline of their life quality and social functions. Long-term follow-up found the different recurrence rate [17], therefore the identification between BOD and SAD is of great clinical importance. In addition, compared with SAD, BOD patients are often accompanied by stenosis of non-relevant intracranial arteries as well as coronary artery or peripheral vascular atherosclerosis [10]. The extra burden of atherosclerosis requires different secondary prevention strategies. The present study reveals that hypertension was more frequently observed in BOD patients when compared to SAD and non-BOD patients, while in older population more hypertension was observed in non-BOD group [10]. Considering the potential relationship between positive vascular remodeling and non-BOD type infarction [3], we speculate that hypertension might tend to induce positive remodeling in older people, although further investigations are needed. Besides statin use [3], control of hypertension might also contribute to prevent BOD type infarction in young patients.

Through comparing the characteristics of stenosis in MCA, this study showed that more than 90% of stenosis in MCA was mild in young BOD patients, which indicates a higher prevalence over that in all age groups reported in literature [10]. On the other hand, non-BOD patients developed mostly severe stenosis or occlusion. Micro-emboli signals are detected through transcranial Doppler examinations for patients with severe intracranial stenosis [18], whereas abnormal perfusion or micro-emboli is rarely detected in BOD patients [19]. In addition, patients with BOD have a more localized MCA stenosis with a location closer to the distal end of the M1 segment than non-BOD patients. However, studies have shown that these two types of stroke have similar atherosclerosis burden [10], indicating that the pathogenesis of BOD may be different from that of non-BOD and BOD may not be a mild form of intracranial atherosclerotic stroke. In terms of therapeutic measures, non-BOD patients may benefit from stenting while BOD patients are not suitable for stent treatment so as to avoid perforating artery occlusion [20]. Classical risk factors such as hypertension, diabetes and smoking are common in young BOD patients. The percentage of intercurrent hypertension particularly exceeds 90%, which is extremely higher than other risk factors, suggesting that hypertension might play a more important role in the pathogenesis of BOD. Further prospective researches are needed to confirm this presumption and the role of risk factors modification in preventing BOD.

This study shows that patients with ischemic stroke induced by intracranial atherosclerosis frequently develop BOD and the clinical manifestations and prognosis are similar to that of SAD. The different features of MCA stenosis suggest that the pathogenesis of BOD and non-BOD patients may be different. BOD, SAD and non-BOD should adopt respective treatment strategy and hypertension control might play a positive role in secondary prevention for BOD. There are several limitations of this study: (1) small number of study subjects may lead to statistical bias; (2) MRA results were used to determine the degree of MCA stenois, however, MRA often overestimate degree of vascular stenosis; and (3) patients with intracranial atherosclerotic plaque may be incorrectly divided into SAD group when MCA habor a stenosis of extremely low degree or no stenosis. Therefore, further large-scale prospective studies with use of high-resolution MRI and CT angiography or catheter angiography are required to confirm the relevant conclusions.

## Conclusions

In conclusion, BOD in young patients with ischemic stroke induced by intracranial atherosclerosis is not rare (33.3%) and its clinical manifestations and prognosis are similar to those of SAD. This may be related to the mild localized stenosis at the distal end in the M1 segment of MCA. Control of hypertension may play a positive role in secondary prevention.

## Abbreviations

BOD: Branch occlusive disease
CAD: Coronary artery disease
DWI: Diffusion weighted imaging
ICAS: Intracranial atherosclerotic stroke
IQR: Interquartile range
LDL-C: Low density lipoprotein-cholesterol
MCA: Middle cerebral artery
MRA: Magnetic Resonance Angiography
MRI: Magnetic Resonance Imaging
NIHSS: National Institutes of Health Stroke Scale
non-BOD: non-branch occlusive disease
SAD: Small artery disease
TC: Total cholesterol
TG: Triglyceide
TOAST: Trial of Org 10,172 in Acute Stroke Treatment
UE: Undetermined etiology
